# Corneal abscess due to *Moraxella nonliquefaciens*

**DOI:** 10.1099/jmmcr.0.005150

**Published:** 2018-04-20

**Authors:** Fernando Cobo, Javier Rodríguez-Granger, Antonio Sampedro, José María Navarro-Marí

**Affiliations:** Department of Microbiology and Instituto Biosanitario, University Hospital Virgen de las Nieves, Granada, Spain

**Keywords:** *Moraxella nonliquefaciens*, corneal abscess, ocular alterations, endophthalmitis, MALDI-TOF MS

## Case summary

A 71-year-old man was seen due to red eye along with loss of vision in the right eye. The patient only referred to a whitish spot on the corneal surface over 3 months probably due to a strange body, but no antimicrobial treatment was started at this stage. Physical examination revealed a central corneal infiltrate in almost all the corneal thickness, an overlying epithelial defect, and a moderate corneal oedema without hypopyon. Several corneal biopsies were taken, and they were directly inoculated to aerobic blood agar (Columbia Agar 5 % Sheepblood, Becton Dickinson), chocolate agar (Choco Agar, Becton Dickinson), Sabouraud agar (Sabouraud Glucose Agar, Becton Dickinson) and thioglycolate broth (Fluid Thioglycollate Medium, Becton Dickinson). All media were incubated at 37  °C, except Sabouraud agar, which was incubated at 30 °C. A corneal biopsy for study of viruses was also taken, being negative for adenovirus, *herpes simplex* (1 and 2) and enterovirus (by polymerase chain reaction). Gram staining of the fluid identified scarce Gram-negative rods. On the first day of incubation the growth of abundant colonies of a non-haemolytic and catalase- and oxidase-positive microorganism was reported in pure culture. No other microorganisms were isolated on the primary plates. A mass spectrometry method (Biotyper, Bruker) was employed to identify the strain as *Moraxella nonliquefaciens* (log score 2.08). The culture in Sabouraud agar was negative after 21 days of incubation.

The MIC of the bacteria to different antibiotics was determined by the E-test method. Until now, no breakpoints have been established for species of *Moraxella* other than *Moraxella catarrhalis.* Taking into account the 2018 EUCAST breakpoints for *M. catarrhalis* [1], the strain was susceptible to all antimicrobials tested, except for amoxicillin (β-lactamase-positive). The MICs were as follows: amoxicillin– clavulanate (0.032 µg ml^−1^), cefotaxime (0.047 µg ml^−1^), levofloxacin (0.06 µg ml^−1^), azythromycin (0.047 µg ml^−1^), thrimetroprim–sulphametoxazole (0.19 µg ml^−1^), and amoxicillin (12 µg ml^−1^).

Treatment was started with vancomycin plus ceftazidime plus cycloplegic eyedrops, along with tobramycin in ointment. Later, vancomycin was stopped and treatment was changed to azythromycin plus ceftazidime eyedrops. The patient responded favourably with slow re-epithelization of the cornea.

**Question**

What is the main cause of endophthalmitis?

**Answer options**

1. Endogenous (bacteraemia or fungaemia).

2. Exogenous (ocular surgery or trauma, extension of corneal infection).

3. Malignant diseases.

4. Idiopatic.

## Discussion

**Correct Answer**: 2. Exogenous (ocular surgery or trauma, extension of corneal infection).

Ocular infections due to *Moraxella nonliquefaciens* have been rarely described. To our knowledge, only eight cases of endophthalmitis due to this microorganism have been previously described in the medical literature [2–7], and here we describe the first case of corneal abscess caused by this pathogen (see Table 1). Most cases of endophthalmitis are exogenous and they are produced as a consequence of ocular surgical procedures, eye traumas or as an extension of corneal infection. Coagulase-negative staphylococci are the most common causes of post-cataract endophthalmitis, and *Bacillus cereus* is a major cause of post-traumatic endophthalmitis.

**Table 1.** Main characteristics of ocular infections due to *Moraxella nonliquefaciens*

**Patient** **(reference/year of publication)** **Author**	**Age (years)/sex**	**Localization**	**Underlying conditions and/or risk factors**	**Clinical manifestations**	**Microbiological diagnosis**	**Identification method**	**Treatment**	**Outcome**
1(/1982) Ebright JR	62/M	Endophthalmos	Cornea scratched by contact lens Cataract removed three years previously Treatment with prednisone and azathioprine (renal transplant)	Scratchy sensation Decrease in visual acuity Injection of the cornea	Vitreous fluid culture	Genetic transformation assay	Gentamicin+cephaloridine Penicillin G	Cure
2(/1985) Lobue TD	67/M	Endophthalmos	Trabeculectomies five years previously Intracapsular cataract extraction in both eyes (six months and one year previously)	Tearing and swelling of the right eyelid Decreased visual acuity, pain, hypopyon, pus Corneal microcystic edema	Vitreous fluid culture	NR	Gentamicin+clindamycin Cefazolin	Cure
3(/1985) Lobue TD	62/F	Endophthalmos	Bilateral trabeculectomies 15 months previously DM	Pain, decreased vision, swelling of the eyelid, hypopyon, pus	Vitreous fluid culture	NR	Gentamicin+cephaloridine+ cefazolin+tobramycin	Cure
4(/1993) Sherman MD	70/F	Endophthalmos	Cataract extraction and trabeculectomy for lens-induced glaucoma five months previously	Progressive pain, redness, photophobia, decreased vision	Vitreous fluid culture	NR	Cefazolin+tobramycin Cefuroxime	Residual ischemic damage
5(/1993) Schmidt ME	79/M	Endophthalmos	Extracapsular cataract extraction and trabeculectomy two months previously	Blurred vision, eye pain, corneal oedema, small hypopyon	Vitreous fluid culture	Biochemical tests	Amikacin+vancomycin+ cefazolin+gentamicin+ceftriaxone	Lost of vision
6(/2002) Laukeland H	78/M	Endophthalmos	Previous trabeculectomy and cataract surgery	Purulent discharge, decreased visual acuity, corneal oedema	Anterior chamber fluid culture	Phenotypic characteristics+16S rRNA	Vancomycin+gentamicin+cefuroxime	Lost of vision
7(/2002) Laukeland H	76/M	Endophthalmos	Cataract surgery and trabeculectomy	Acute blurred vision, purulent discharge, corneal oedema	Vitreous fluid culture	Phenotypic characteristics+16S rRNA	Vancomycin+gentamicin+cefuroxime	Lost of vision
8(PR/2017) Cobo F	71/M	Cornea	Corneal damage	Red eye, loss of vision, corneal oedema	Corneal abscess culture	Maldi-tof MS	Vancomycin+ceftazidime+tobramycin Azythromycin	Improved

**M:** male; **F:** female; **DM:** diabetes mellitus; **NR:** not reported; **CRP:** C-reactive protein; **PR:** present report.

The case of Mandelbaum *et al.* [3] did not show sufficient data to be included in the table.

Ocular infections, such as corneal abscesses and endophthalmitis, are a medical emergency. Treatment of corneal traumas is very important in order to avoid dissemination of infection into the eye. Prompt and appropriate treatment of these lesions may help to both avoid complications and recover total vision.
